# The microwave-assisted ionic liquid nanocomposite synthesis: platinum nanoparticles on graphene and the application on hydrogenation of styrene

**DOI:** 10.1186/1556-276X-8-414

**Published:** 2013-10-08

**Authors:** Jer-Yeu Lee, Tung-Yuan Yung, Ling-Kang Liu

**Affiliations:** 1 Department of Chemistry, Academia Sinica, 1 Roosevelt Road Section 4, Taipei 106, Taiwan; 2 Institute of Chemistry, Academia Sinica, 128 Academia Road Section 2, Nankang, Taipei 115, Taiwan; 3 Department of Physics, National Central University, 300 Jhongda Road, Jhongli, Taoyuan 320, Taiwan; 4 Molecular Science and Technology, Taiwan International Graduate Program, Academia Sinica, 128 Academia Road Section 2, Nankang, Taipei 115, Taiwan; 5 Nuclear Fuels and Materials Division, Institute of Nuclear Energy Research, 1000 Wenhua Road, Longtan, Taoyuan 325, Taiwan

**Keywords:** Graphene, Pt nanoparticle, Ionic liquid, Microwave, Hydrogenation of styrene

## Abstract

The microwave-assisted nanocomposite synthesis of metal nanoparticles on graphene or graphite oxide was introduced in this research. With microwave assistance, the Pt nanoparticles on graphene/graphite oxide were successfully produced in the ionic liquid of 2-hydroxyethanaminium formate [HOCH_2_CH_2_NH_3_][HCO_2_]. On graphene/graphite oxide, the sizes of Pt nanoparticles were about 5 to 30 nm from transmitted electron microscopy (TEM) results. The crystalline Pt structures were examined by X-ray diffraction (XRD). Since hydrogenation of styrene is one of the important well-known chemical reactions, herein, we demonstrated then the catalytic hydrogenation capability of the Pt nanoparticles on graphene/graphite oxide for the nanocomposite to compare with that of the commercial catalysts (Pt/C and Pd/C, 10 wt.% metal catalysts on activated carbon from Strem chemicals, Inc.). The conversions with the Pt nanoparticles on graphene are >99% from styrene to ethyl benzene at 100°C and under 140 psi H_2_ atmosphere. However, ethyl cyclohexane could be found as a side product at 100°C and under 1,520 psi H_2_ atmosphere utilizing the same nanocomposite catalyst.

## Background

Catalysts using metal nanoparticles have been one of the most interesting research areas in recent years since its relevance to chemical [1-4], pharmaceutical [5-8], and energy-related applications [9-11]. Recently, some researchers have shown that nanocatalysts with high dispersion and narrow size distributions stabilized by appropriate supports or capping materials can work under mild conditions with high activity and high selectivity when compared to conventional heterogeneous catalysts. It is known that the transition metal nanoparticles are effective catalysts, in which the shape, size, and surface structure of the solid supports all that contribute to the catalytic activity [1-4,9-13]. The supports usually are alumina, zeolite, and carbon materials that further include the carbon black, carbon nanotubes, graphene, and nanoporous carbon [14-20].

**Figure 1:**
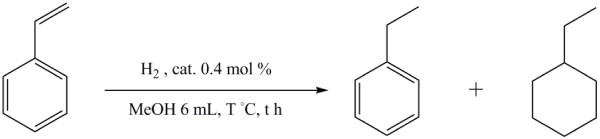
The hydrogenation reaction of styrene to ethylbenzene and ethylcyclohexane.

Graphene is the most important and eye-catching carbon material since 2004 [21]. The graphene as catalyst support is known with many applications, such as in catalysis, in photodevices, and in enhancing electronic property [22-24]. Conventionally, the synthesis of metal nanoparticles on graphene follows the methods of polyol reduction, hydrothermal and solvothermal synthesis, and CVD, etc. [21-24]. In this study, we employed a simple method to synthesize the nanocomposite, abbreviated as Pt/GE and Pt/GO, in that the Pt precursor was dissolved in just the ionic liquid of 2-hydroxyethanaminium formate [HOCH_2_CH_2_NH_3_][HCO_2_], without any additional organic solvents or any additional reducing agents in the system. And this method was further microwave-assisted so that the synthesis was more efficient in time and less wasting in energy. The total synthesis was accomplished under 20 min. The centrifugation for purification was, however, the main time-consuming step yet to improve. The samples were applied later on the hydrogenation of styrene (Figure 1) for a comparison with results from the commercially available activated carbon-supported Pd and Pt catalysts, Pt/C and Pd/C.

## Methods

The synthesis of graphite oxide and graphene followed the well-known Hammer's method [25]. A 250-mL round bottomed flask filled with 25 mL concentrated sulfuric acid (98%, Adrich, St. Louis, MO, USA) was held in an iced bath. After 5 to 10 min, 10 mL fumed nitric acid was added slowly in 15 min. Then, graphite powder (1.0 g, with particle size <45 μm) was added into the mixture under vigorous stirring for 30 min with the flask held in the iced bath. Then 22 g potassium chlorate was added into the solution in 30 min, and the mixture was stirred at room temperature for 96 h. The solution was centrifuged with a suitable amount (about 200 to 300 mL) of deionized (DI) water added under an iced bath temperature. Removal of liquid phase, followed by addition of DI water and then centrifugation, was repeated for three times. The mud-like residue was dried at 80°C for 12 h to produce the graphite oxide.

The nanocomposite synthesis followed a procedure similar to that reported in our previous study [26]. Graphite oxide (250 mg) was added in 250 mL DI water and stirred for 30 min before addition of 1.4 g NaBH_4_, and the mixture was kept at 80°C for 1 h. Prior to sulfonation, the solution was centrifuged for collection of residues that were rinsed with methanol for three times then dried at 80°C under the N_2_ atmosphere for 1 h. The graphite oxide was sulfonated and exfoliated to graphene with the following procedure: in a 500-mL round-bottomed flask, the residues (158 mg) in 300 mL DI water were dispersed using an ultrasonic bath for 30 min. Separately, sulfanilic acid (140 mg) and potassium nitrate (50 mg) were introduced into a 100-mL beaker containing DI water (40 mL) employing an iced bath. After being mixed well, the solution was added with 1 N HCl (1 mL) and then the solution was poured into the above mentioned round-bottomed flask and stirred for 2 h in the iced bath. Centrifugation followed by removal of aqueous solution resulted in the sulfonated graphene, which was rinsed with methanol for a few times then dried at 80°C under the N_2_ atmosphere.

The microwave-assisted synthesis of Pt/GE and Pt/GO was performed using a CEM Discover Du7046 microwave set (Matthews, NC, USA) with 80 W power output for 30 s then held at 80°C for 5 min. The nanocomposites were prepared with sulfonated graphene or graphite oxide (100 mg) as substrates together with grinded K_2_PtCl_6_ at 14.5, 355, or 15 mg, respectively, plus 2-hydroxyethanaminium formate (5.0 g), in Pyrex glass tubes (results shown in Table 1). The preparation of 2-hydroxyethanaminium formate was in term by a slow neutralization of H_2_NCH_2_CH_2_OH (20 mL) and formic acid (14 mL) in a 100-mL round-bottomed flask kept in an iced bath [27].

**Table 1:** **Synthesis of the nanocomposites in ionic liquid 2**-**hydroxyethanaminium formate with microwave assistance**

	**Loading (mg)**			
**Entry**	**K**_**2**_**PtCl**_**6**_	**Ionic liquid**	**Substrate (100 mg)**	**Shape/Size (nm)**
1 (Pt/GE)	14.5	15000	Graphene	Sphere/14 ± 6
2 (Pt/GO)	100	15000	Graphite oxide	Cube-like/18 ± 8
3 (Pt/GO)	15	15000	Graphite oxide	Cube-like/4 ± 7

The analytical instruments used were as the following: nuclear magnetic resonance (NMR) with Bruker AVA-400, Madison, WI, USA (400 MHz), element analysis (EA) by FLASH EA 1112 Series, Thermo Finnigan, Milano, Italy, X-ray diffraction (XRD) by Phillips PANalytical X'Pert PRO MPD, Amsterdam, The Netherlands (Cu, *λ* = 0.1541 nm, 2 theta: 5° to 80°), thermal gravity analysis (TGA) with Perkin Elmer 1 TGA, Waltham, MA, USA (2 to 5 mg samples in Pt plate with 5°/min heating rate), transmitted electron microscopy (TEM) with JEOL JEM-2010, Akishima-shi, Japan (LaB6, 200 kV), gas chromatography (GC) by Agilent Technologies 7890A GC system with Agilent Technologies 7683B Series injector, Santa Clara, CA, USA.

The hydrogenation of styrene was performed with a Parr 4762 (Q)* reactor, Moline, IL, USA, under two H_2_ pressure conditions: one at 100°C under 1,520 psi and the other at 100°C under 140 psi H_2_ atmosphere, both with a reaction time of 1 h. The hydrogenation of styrene with commercial Pd/C was loaded with catalyst 50 mg and styrene 1.22 g then 6 mL methanol was added in the Parr 4762 (Q)* reactor. Similar hydrogenation with commercial Pt/C was loaded with 50 mg of catalyst and 667 mg of styrene followed by 6 mL methanol in the reactor. For model catalyst (Pt/GE) experiments, it was added in the 4762 (Q)* reactor with 20 mg catalyst and 320 mg styrene with 6 mL methanol. After hydrogenation, the reactor was cooled down to room temperature; the mixed hydrogenation products were filtered with diatomite, and the liquid phases were analyzed with GC.

## Results and discussion

**Figure 2:**
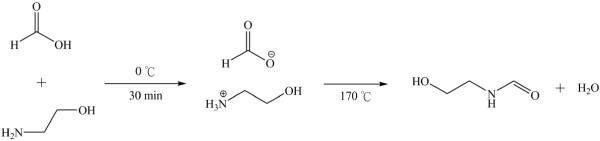
The synthesis of ionic liquid of 2-hydroxyenthanaminimium formate and the thermal transformation.

**Figure 3:**
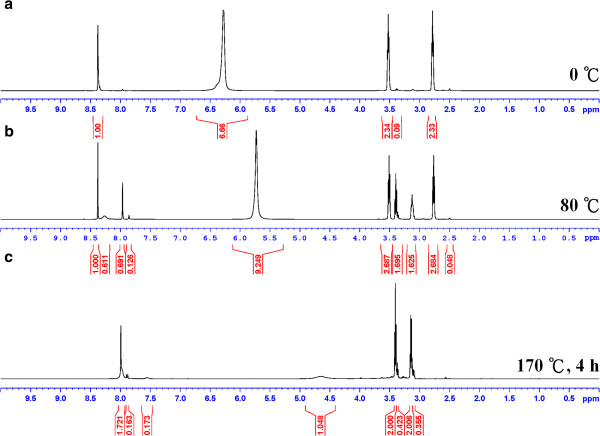
^**1**^**H NMR spectra of 2**-**hydroxyethanaminium formate synthesized. (a)** At 0°C, **(b)** at 80°C, and **(c)** the resultant^1^H NMR from **(a)** upon heating at 170°C for 4 h.

The ionic liquid 2-hyroxyethanaminium formate was prepared at low temperature by a slow neutralization reaction between 2-hyroxyethanamine and formic acid in exact 1:1 molar ratio (Figure 2). The temperature at which neutralization was performed is important because only when the ionic liquid was made at temperature strictly lower than 0°C that the ^1^H NMR results exhibit a spectrum consistent to the formula of [HOCH_2_CH_2_NH_3_][HCO_2_], as shown in Figure 3a. The heat released during neutralization should be carefully controlled at minimal to keep side reactions to occur that lead to 2-hyroxyethyl formamide or 2-aminoethyl formate (see Figure 3b,c).

**Figure 4:**
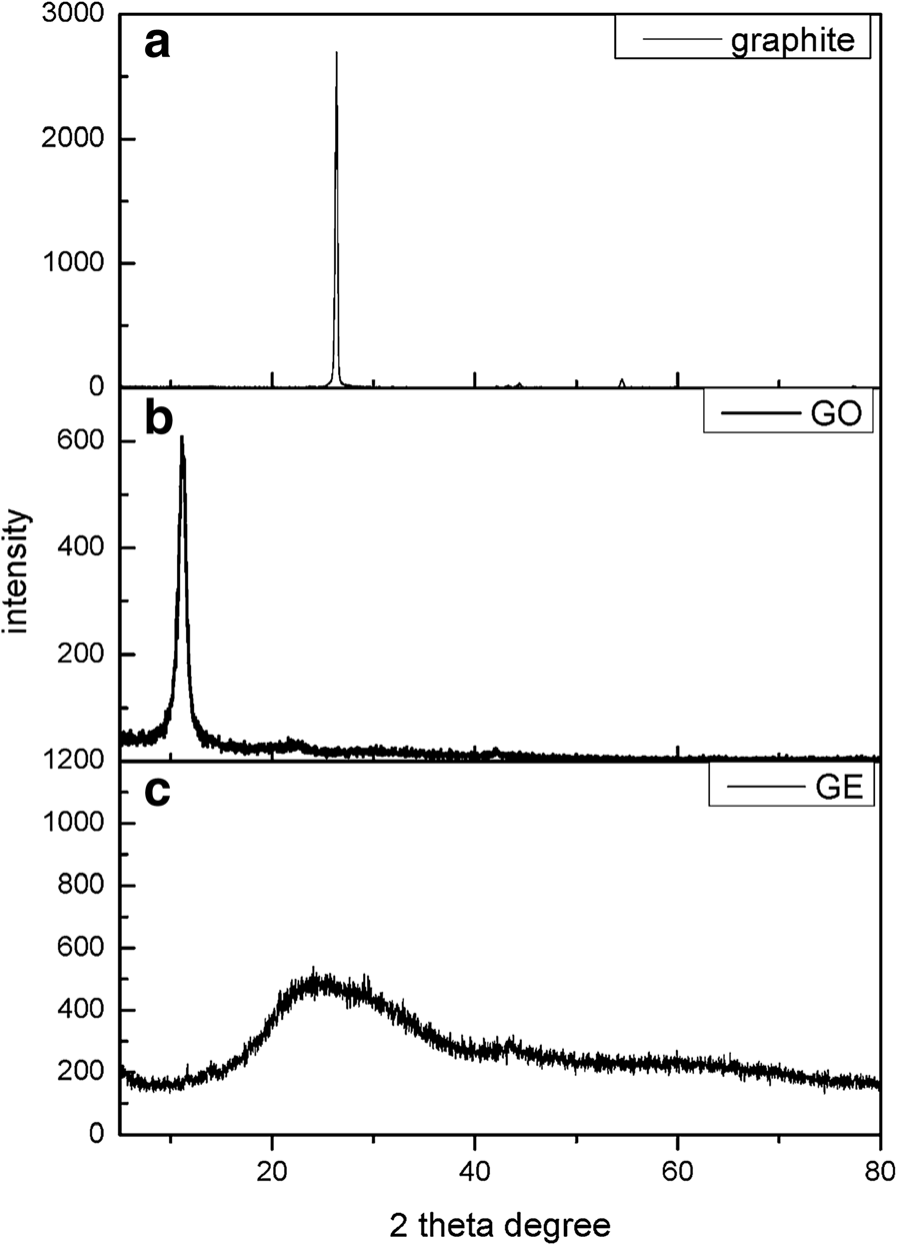
The XRD patterns for (a) graphite as received (graphite), (b) graphite oxide (GO), and (c) graphene (GE), respectively.

The XRD analysis of the substrates was shown in Figure 4, and the elemental analysis of nanocomposites was shown in Table 2. The nanocomposite synthesis was controlled at 80°C, at which a portion of the ionic liquid could have been transformed to 2-hyroxyethyl formamide in addition to the main function to convert the Pt precursor to Pt nanoparticles; the ionic liquid being a solvent and a sacrificing reductant.

**Table 2:** **The EA results of graphite oxide**, **sulfonated**-**graphite oxide and graphene**

**Sample**	**C wt%**	**H wt%**	**N wt%**
GO	32.98	2.40	-
GO-SO_3_H	44.62	2.47	1.04
GE	61.82	2.11	2.4

**Figure 5:**
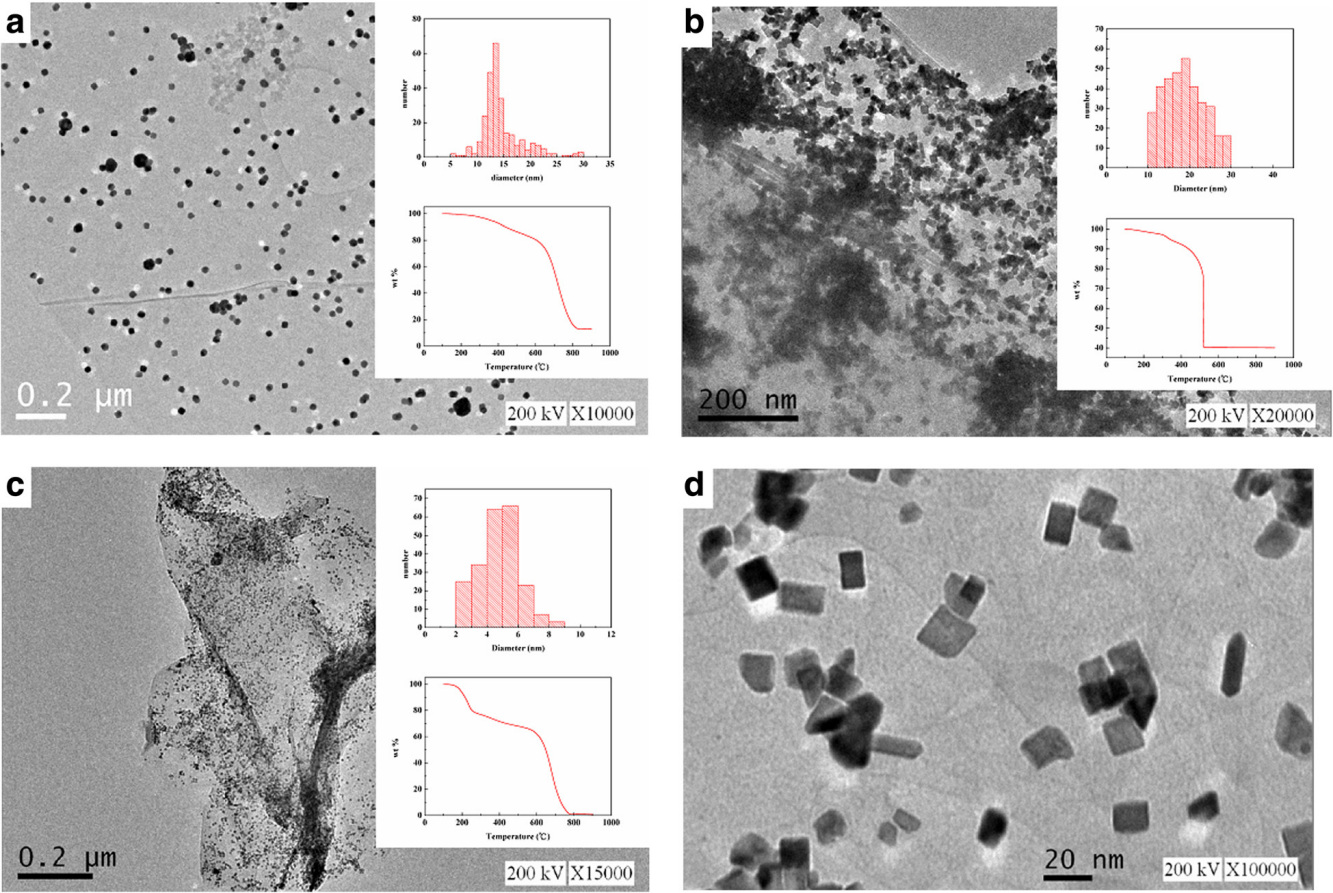
**The TEM morphologies of the nanocomposites. (a)** Entry 1, 12 wt.% Pt loading on graphene, **(b)** entry 2, 40 wt.% Pt loading on graphite oxide, and **(c)** entry 3, 14 wt.% Pt loading on graphite oxide, **(d)** cube-like morphology of entry 2 with × 100,000 magnification. The upper intersectional images are the particle size distributions, and the lower intersectional images are the TGA results.

The analysis of morphology and particle size distribution was done by TEM, as shown in Figure 5. In Table 1, entry 1 was found to have sphere morphology with 14.6 nm average particle size and the Pt loading was 12 wt.% from TGA results. And entries 2 and 3 were with 40 wt.% and 14 wt.% in Pt loading and were with 18.8 and 4.7 nm in average particle sizes, respectively. With similar Pt precursor to ionic liquid ratio (entries 1 and 3), the nanocomposites produced with the graphite oxide substrate have much smaller Pt particle sizes and more Pt particles loading (approximately 14 wt.%) when compared to those produced with the graphene substrate (approximately 12 wt.%). Our previous study showed also that the particle size distribution for Pt loading at 63 wt.% on graphene was about 6 ± 3 nm [26]. The shapes of Pt nanoparticles on graphite oxide were cubic-like in the current study. We supposed that on the surfaces of graphite oxide are more oxygen-functional groups in favor of anchoring the Pt precursors and formation of the cubic-like shape nanoparticles. On the contrary, on the surfaces of graphene, the oxygen functional groups are much less than that on the surfaces of graphite oxide. Thus, at the same Pt loading, the two substrates would not produce the same shapes and sizes of Pt nanoparticles on graphite oxide or on graphene. But in our previous study of 63 wt.% Pt loading, we did synthesize the cubic Pt on graphene [26]. Herein, the hydrogenation of styrene was examined using the same weight percentage of Pt loading.

From the literature survey, CNT-supported palladium (Pd/CNT) and gold (Au/CNT) nanoparticles show negligible catalytic activity for the hydrogenation of benzene at room temperature. Using the Pd/CNT catalyst at 50°C with 10 atm H_2_, a conversion of benzene to cyclohexane (48.8% after 24 h) was observed. Rh/CNT and Pt/CNT show good catalytic activities for the hydrogenation of benzene at room temperature with ≥50% conversion to cyclohexane in 24 h. Cyclohexane is the only product detected in the hydrogenation of benzene [28], suggesting that the partially hydrogenated intermediates were only transient. The hydrogenation of styrene, employing the current nanocomposites Pt/GE, was on the side chain instead. The hydrogenation after 1 h could convert >99% of styrene to ethylbenzene. Benzene hydrogenation is an ideal reaction for such studies as it has been investigated extensively on single-crystalline Pt surfaces. Because this reaction has been shown to produce only cyclohexane on Pt(100) and both cyclohexene and cyclohexane on Pt(111), thus, suitable for probing nanoparticle shape-dependent reaction selectivity in catalysis [27]. The Pd, Pt, and Ru species were investigated on the *γ*-Al_2_O_3_ supported catalysts for hydrogenation of styrene, and the group VIII metals were the best choices. The hydrogenation of styrene activity of metal catalysts on the supported alumina material followed the order Pd > Pt > Ru [29].

Also, the benzene hydrogenation catalytic activity of the CNT-supported metallic nanoparticles increases in the order Pd/CNT < Au/CNT < Rh/CNT < Pt/CNT < Pd-Rh/CNT. For the CNT-supported single metallic nanoparticle catalysts, this order follows generally the same trend as the typical catalytic activities of transition metals known for hydrogenation of benzene, i.e., Co < Pd < Ni < Pt < Ru < Rh [11]. The reason for this order is not known in the literature, but the solvent has been shown to play a role on the hydrogenation of monocyclic arenes in the conventional heterogeneous catalytic system using transition metals as catalysts. The difference in enthalpy of vaporization among the transition metals has also been related to their difference in catalytic activity [11]. The hydrogenation results of Pt/GE nanocomposites were shown in Table 3.

**Table 3:** **The results for hydrogenation of styrene from Pt/G and commercial catalysts**

	**Metal (wt%)**	**Size (nm)**	**Reaction condition**	**Product (%)**		
				**Styrene**	**Ethylbezene**	**Ethycyclohexane**
Pt/GE	12	14.6	100°C,140 psi,1 h	3.21	96.79	-
Pt/C	10	2 ~ 5	Same	-	>99	-
Pd/C	10	3 ~ 5	Same	-	>99	-
	Metal (wt%)	Size (nm)	Reaction condition		Product (%)	
				Styrene	Ethylbezene	Ethycyclohexane
Pt/GE	12	14.6	100°C,1520 psi,1 h	-	99.66	0.34
Pt/C	10	2 ~ 5	Same	59.69	40.31	-
Pd/C	10	3 ~ 5	Same	-	99.87	0.13

## Conclusions

The low H_2_ pressure hydrogenation reaction condition exhibited a catalytic activity in the order Pd/C to Pt/C > Pt/GE. However, the high H_2_ pressure hydrogenation reaction condition gave an order of Pd/C > Pt/GE > Pt/C. The hydrogenation activity of Pt/GE was better than the commercial Pt/C but a little less than that of the commercial Pd/C.

## Competing interests

The authors declare that they have no competing interests.

## Authors’ contributions

LJU collected and analyzed the data and organized the figures. YTY organized and wrote the content of manuscript. LLK supervised the project and corrected the paper. LKL and YTY are the corresponding authors. All authors read and approved the final manuscript.
